# Comparative Analysis of Cyclic Properties and Fractography of AA2519 Aluminum Alloy Samples with Different Fatigue Lives

**DOI:** 10.3390/ma18215021

**Published:** 2025-11-04

**Authors:** Robert Sołtysiak, Maciej Kotyk, Joanna Małecka

**Affiliations:** 1 Faculty of Mechanical Engineering, Bydgoszcz University of Science and Technology, Al. prof. S. Kaliskiego 7, 85-796 Bydgoszcz, Poland; maciej.kotyk@pbs.edu.pl; 2 Faculty of Mechanical Engineering, Opole University of Technology, ul. Prószkowska 76, 45-758 Opole, Poland; j.malecka@po.edu.pl

**Keywords:** AA2519, cyclic properties, stress-strain curve, Ramberg-Osgood, full-range curve

## Abstract

The article presents an analysis of the outcomes for AA2519 aluminum alloy exposed to variable loads. The variable loads were implemented with a strain control program consisting of incremental steps and increasing/decreasing multiple steps. Tests were conducted at higher and lower strain ranges and yielded lower (LFL test) and higher (HFL test) fatigue life, respectively. The values of plastic strain, cyclic modulus, cyclic yield strength, and fractography were analyzed. Based on the analysis of the test results, a criterion was established for the division of the tested fatigue properties into two parts for which the strength coefficient and strain hardening exponent were determined. An analytical description of the cyclic stress–strain curve for the entire range of results obtained from the LFL and HFL tests was proposed. Compared to other available models describing material properties, good compliance was obtained with the experimental results for both the LFL and HFL tests.

## 1. Introduction

Due to their relatively high strength, good fracture toughness, and relatively low density, AA2519 alloys are used in ballistic structures as well as in lightweight military [1,2], aviation [3], and space [4] structures. The above-mentioned structures are very often exposed to time-varying loads, thus the knowledge of the behavior of this type of material under time-varying conditions becomes indispensable when designing such structures.

Available studies on fatigue tests of the AA2519 alloy are very scarce. The study [5] analyzed the effect of rolling direction on static and fatigue properties and demonstrated that rolling direction does not significantly affect fatigue properties. Another study, ref. [3] compared the fatigue life results of the AA2519 alloy with those of other 2000-series alloys. Most of the studies on fatigue testing of AA1519 alloy involve tests where the alloy is joined with another material, mainly by explosive welding [6,7,8]. Results of the aforementioned studies are mainly related to normative tests [9,10] at constant-amplitude loads with the exception of studies [6] where tests were carried out using an incremental-step method. Those studies highlighted that the AA 2519 alloy is cyclically unstable and subject to cyclic hardening.

Normative studies [9,10] are costly and labor-intensive. Methods for the determination of cyclic properties with improved cost- and time-efficiency have been introduced in recent years. These are methods based on single specimens [11,12], for which similar test results are obtained (e.g., [13]) compared to those obtained using standard methods. Moreover, study results [14] are available, which prove that in the case of estimating fatigue life of randomly loaded structural elements, the method of determining cyclic material properties using the single specimen method makes it possible to obtain more reliable fatigue life results.

Geometric notches of such structures, which are caused, among other things, by changes in cross-section or by mounting holes, trigger stress/strain concentrations. The strain/stress values at the bottom of the notch of a structural member are determined using different types of constitutive model of cyclic properties of the material [15,16]. Nevertheless, in engineering practice, the most commonly used model for representing the cyclic stress–strain curve (CSS) is the Ramberg–Osgood (R–O) model [17,18], which consists of the elastic and the plastic parts.

Research on the development of material models that will better describe results of experimental tests are still ongoing. Thus, the R–O model has undergone several modifications, which, depending on the type of material, provide better or worse compliance with experimental results. The elastic part of the R–O model itself, described by Young’s modulus, becomes debatable. There are at least three types of moduli for static tests: Young’s Modulus, Tangent Modulus, and Chord Modulus [19]. In addition, researchers specify an effective Young’s modulus [20] as the slope of the first elastic response in the stress–strain diagram. Others introduce cyclic moduli defined for the increasing loop branch and for the descending loop branch, and a cyclic modulus defined as the slope of a straight line connecting the loop corners [21,22].

Such a large number of moduli raises the question of which modulus is the most appropriate for use in cyclic properties. Some argue that the cyclic modulus should be determined within the elastic limit of the first quarter of the hysteresis loop [21], others that the mean value of cyclic moduli determined from the increasing and descending branches of the hysteresis loop should be used [23,24], but some of them [24] limit the range of averaging the cyclic modulus to a certain value of the strain, thus obtaining good results.

Some researchers, when describing the experimental results of the cyclic stress–strain curves introduce additional material constants that fit the model to the experimental results of a particular material [25], obtaining very good results. Others propose two-stage Ramberg–Osgood models [24,26], achieving better model fit to experimental results.

Descriptions of full-range stress–strain curves have also appeared in recent years, but not all of them describe the entire range of the stress–strain diagram using a single formula. In the study [27] the range has been divided into two parts and the description was based on conventional parameters of the Ramberg–Osgood model and ultimate tensile strength (S_u_) and strain (ε_u_). In [28], full-range stress–strain curves are divided into two parts. There is also a model that divides the stress–strain curve into three stages [29], and the determination of all necessary parameters is based on four basic data (initial elastic modulus, 0.2% proof stress, ultimate stress, and total strain of ultimate point). This model is based on a modification of the Hradil model [30]. In [31], full-range stress–strain curves were described by one comprehensive formula which is based on conventional R–O parameters and two additional material parameters, A and B, were introduced. A slight modification of this model was proposed in [32] to describe the monotonic properties in individual zones of the laser weld of duplex steel. Good compliance with experimental results was obtained here.

The slight modification of the model describing the entire range of the stress–strain curve proposed in [32] did not include a criterion for dividing the experimental data for which n_1_, K_1_, n_2_, and K_2_ constants were determined. The division of these experimental data was carried out intuitively. This paper proposes a criterion for dividing experimental tests based on the intersection points of linear correlations describing cyclic moduli as a function of plastic strain amplitude and linear correlations of control strain amplitude as a function of plastic strain amplitude. Based on this criterion, the cyclic material properties of AA2519 alloy were described using a modified model describing the full-range stress–strain curve. A very good level of compliance between the proposed model and experimental results of the LFL and HFL test for AA2519 aluminum alloy subjected to time-varying loads was obtained using the above criterion.

## 2. Materials and Methods

Research on selected cyclic material properties and microstructure was carried out on the relatively new aluminum alloy AA2519. Prior to tests, the prepared specimens were subjected to heat treatment involving soaking at a temperature of 530–550 °C for 2 h, cooling in water with rate > 150 °C/s (in the range of 500–300 °C), and aging at 165 °C for 10 h (heat treatment state T62). The heat treatment was intended to improve mechanical properties of the tested alloy. Chemical composition of this alloy and properties obtained from the static tensile test are presented in Table 1.

**Figure 1:**
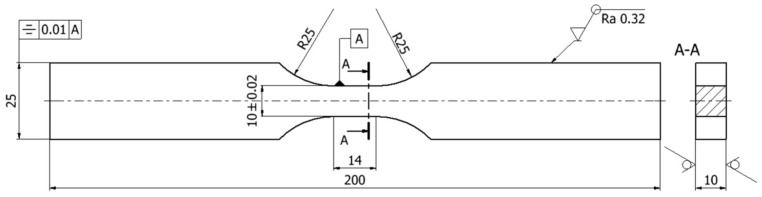
Dimensions of the specimen (in mm) for testing cyclic fatigue properties.

Tests of main fatigue properties were carried out for specimens taken from a 10 mm thick sheet metal plate. Specimens were cut parallel to the rolling direction using the WEDM method. Dimensions of specimens are shown in Figure 1.

Tests of fatigue properties were performed in low-frequency range of cycles using the INSTRON 8502 testing machine (Bydgoszcz University of Science and Technology, Bydgoszcz, Poland). The fatigue test was controlled by the strain value. The tests were carried out using an extensometer with a 10 mm measuring base and a measuring range of +/−1 mm. The tests were carried out at a frequency of 0.2 Hz and a cycle asymmetry ratio of R = −1.

**Figure 2:**
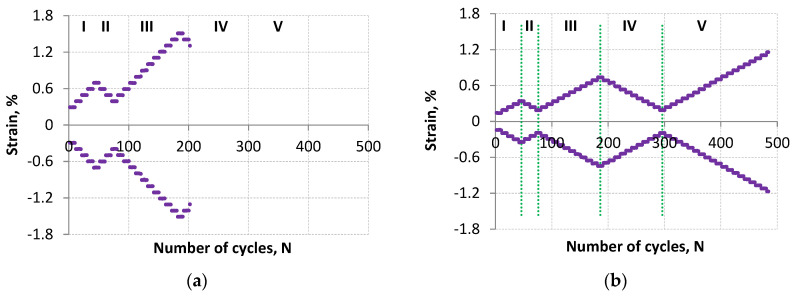
Specimen loading program for lower fatigue life (LFL) (**a**) and for higher fatigue life (HFL) (**b**).

The loading program was designed as a strain control program consisting of incremental steps and increasing/decreasing multiple steps described in [12]. The load was imposed by controlling the strain by alternating multiple steps with increasing strain (step I, III, and V—Figure 2) and multiple steps with decreasing strain (step II and IV—Figure 2). Thus several strain levels were implemented in each block by performing five cycles at each strain level.

This combination of the programs was chosen because the stress–strain curves obtained from the incremental step test provide the best strain/stress response to random loading occurring in objects made of the tested material. Combining the incremental step with the multiple step (e.g., using five cycles at a given level) provides a greater chance for the material to stabilize at a given level, thus obtaining closed hysteresis loops, which improves data processing for stress–strain curves.

The tests were carried out using two specimens for two different loading programs. These programs were of the same nature but differed in the strain range values. For example, the first loading program—step I (Figure 2a) started with a strain range of Δε = 0.6% and ended with Δε = 1.4%, while the second loading program—step I (Figure 2b) started with a strain range of Δε = 0.3% and ended with Δε = 0.7%. Thus, it should be noted that the second loading program was characterized by half the smaller control strain ranges Δε compared to the ranges of the first loading program. Reduction in the strain range increased the fatigue life of the test specimen.

Throughout the tests, the outcomes in the form of points of the hysteresis loop were recorded. Tests were conducted up to complete fatigue fracture, obtaining different fatigue lives of the tested items. After the fatigue tests, photographs of fatigue fractures were taken with the SEM and analyzed. An analytical description of material properties was also proposed on the basis of the developed results.

## 3. Results and Discussion

### 3.1. Cyclic Stress Response

**Figure 3:**
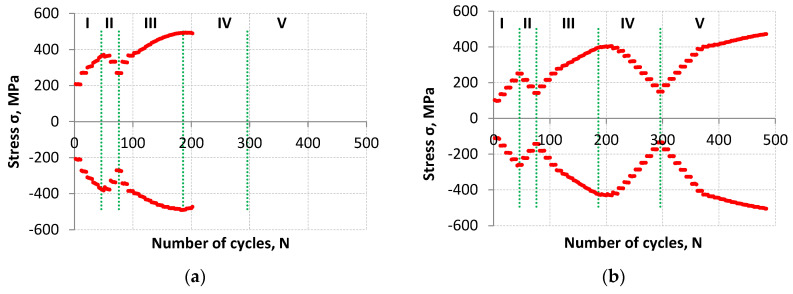
The maximum and minimum stress values as a function of cycles for the lower fatigue life (**a**) and for the higher fatigue life (**b**).

Fatigue test control with the strain value (Figure 2) made it possible, among other things, to record the response in the form of maximum and minimum stress values (Figure 3). Regardless of the fatigue life obtained, the amplitude of stress preceding the total fracture of the specimens was similar. For LFL, this amplitude was 482 MPa (for a maximum value of 490 MPa and a minimum value of −474 MPa), while for the higher fatigue life it was 488 MPa (for a maximum value of 472 MPa and a minimum value of −504 MPa).

The highest value of the stress amplitude was achieved for the LFL specimen in the 186th cycle and it amounted to σ_a_max_LFL_ = 491 MPa while for the HFL specimen in the 484th cycle and it amounted to σ_a_max_HFL_ = 488 MPa. In both cases, these values were recorded at the same control strain amplitude value of ε_a_ = 1.5%. Both σ_a_max_ values are higher than the material strength S_u_ = 475 MPa obtained in the tensile test by about 3.4% and 2.8%, respectively. Higher stress amplitude values in cyclic tests than the material strength indicate cyclic hardening of the material.

**Figure 4:**
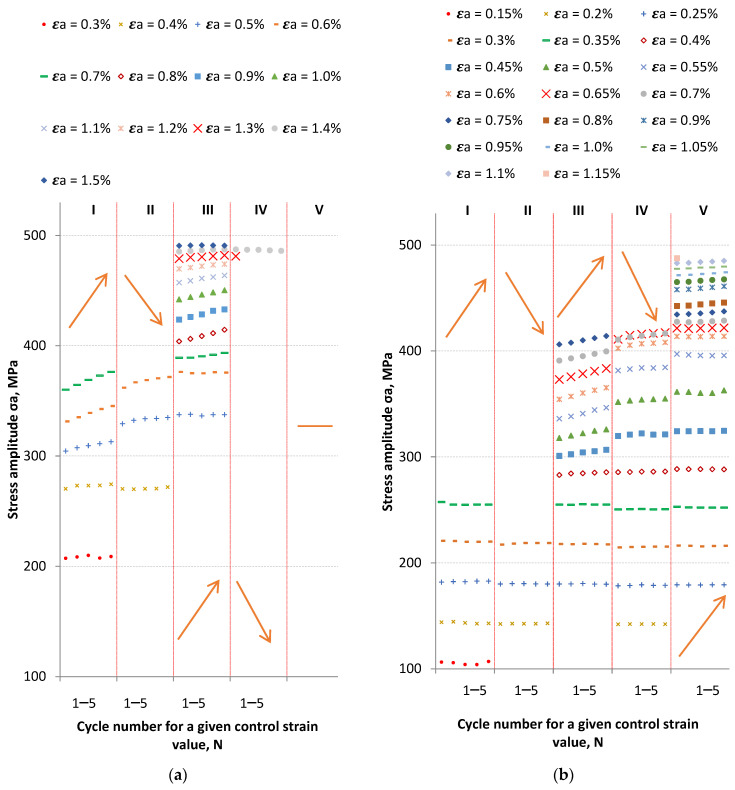
The graph of Δσ/2 as a function of the number of cycles for LFL (**a**) and HFL (**b**).

In order to analyze the behavior of the material at given levels of control strain values, the values of stress amplitudes as a function of the number of cycles were compared. The number of cycles was assumed from 1–5, since the control strain changed every five cycles. The results for LFL are summarized in Figure 4a, while those for HFL are shown in Figure 4b. Moreover, the up arrow indicates multiple steps with increasing strain while the down arrow indicates multiple steps with decreasing strain.

At the lowest levels of control strain (ε_a_ = 0.3–0.4% for LFL and ε_a_ = 0.15–0.35% for HFL), the material was relatively stable. A slight weakening can be observed for LFL for ε_a_ = 0.4 at the turn of step I and step II, while for HFL the weakening progressed gradually and was virtually invisible in graph 5b. For example, at a strain of ε_a_ = 0.25%, the stress amplitude σ_a_ for the first cycle at step I was 181.9 MPa, and for the last cycle at step V, 179.36 MPa. While at a control strain of ε_a_ = 0.3%, the stress amplitude was 220.84 and 216.23 MPa, respectively.

For both LFL and HFL, starting from the control strain value of ε_a_ ≈ 0.45–0.5%, the material began to harden. For LFL, the greatest hardening was observed for control amplitude of ε_a_ = 0.6%. For this loading level, a stress amplitude of 331.37 MPa was recorded for the first cycle of step I. While for the first cycle of step III, the stress reached 375.59 MPa. The hardening observed for a control strain of ε_a_ = 0.6% was thus approximately 13%. At a comparable control strain of ε_a_ = 0.55%, higher hardening was observed for the HFL test results. For the first cycle of step III, the stress amplitude was 336.18 MPa, while a stress of 397.21 MPa was recorded for the first cycle of step V. The hardening was therefore over 15%. After this first cycle of the stress amplitude in step V, there was a slight weakening of the material for the same value of control strain. This weakening of the material was also evident in subsequent loading cycles, when the control strain of ε_a_ = 0.55% in Step V progressed to subsequent levels of 0.6, 0.65, and 0.7%.

When analyzing the available test results obtained with strain control under constant-amplitude conditions, hardening (if any) is noticeable mainly in the initial steps of fatigue life. In the last step of fatigue life, the material usually weakens, which was also observed for the LFL tests (V, ε_a_ = 1.4%). However, the behavior of the material in the last step of fatigue life during the HFL test may be debatable. In this case, material hardening occurs virtually until complete fracture.

### 3.2. Mean Stress Relaxation

**Figure 5:**
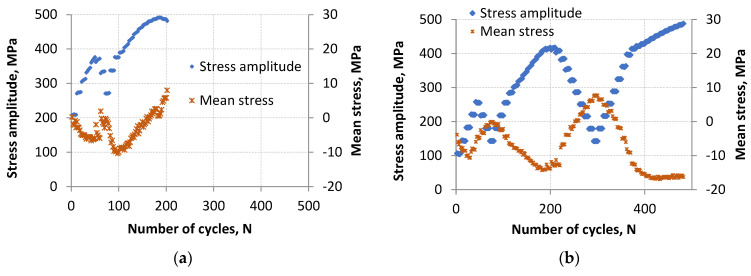
The curve of σa and σm as a function of the number of cycles for LFL (**a**) and HFL (**b**).

The behavior of mean stress values was also analyzed. Graphs of mean stress and stress amplitude as a function of the number of cycles are shown in Figure 5. A change in the value of mean stress as a function of the number of cycles is observed for both LFL and HFL. These changes ranged from −10.39 to 7.97 MPa for LFL and from −17.57 to 8.04 MPa for HFL. Broader range of mean stress changes for HFL reveals the occurrence of more extensive damage mechanisms under the influence of increasing numbers of cycles, which had a positive effect on the hardening of the material virtually until the last cycle before the failure of the specimen.

When analyzing the nature of changes in mean stresses, it should be noted that during most of the fatigue life period, an increase in the value of maximum stress causes a decrease in mean stress values. However, while the maximum stress values decrease, the mean stress values increase. This regularity is observed for both cases (Figure 5a,b). The change in this behavior begins to be observed before exceeding the hundredth loading cycle for LFL and after exceeding the four hundredth cycle for HFL. At that point, the mean stress value began to increase from −10.39 to 7.97 MPa for LFL and from −17.57 to −16.43 MPa for HFL regardless of whether successive load cycles were increasing (as observed for HFL) or decreasing (as observed for LFL). After passing the four hundredth cycle for HFL, the increase in the mean stress value was negligible and amounted to more than 1 MPa while for LFL the change was significant and amounted to as much as 18.36 MPa.

The change in the behavior of mean stress values at the end of fatigue life (LFL test), causing the mean value to shift toward larger values, can be caused by the appearance of large stress concentrations in the areas of developing micro- and macro-cracks, accompanied by large strain necessary to achieve forced control strain. These processes can decrease compression strength of the material and increase its tension strength. Such phenomena are not observed at constant-amplitude loads. On the other hand, the stabilization of mean values (HFL test) may indicate that cyclic equilibrium has been reached in the material in which stresses and microstructure have stabilized.

**Figure 6:**
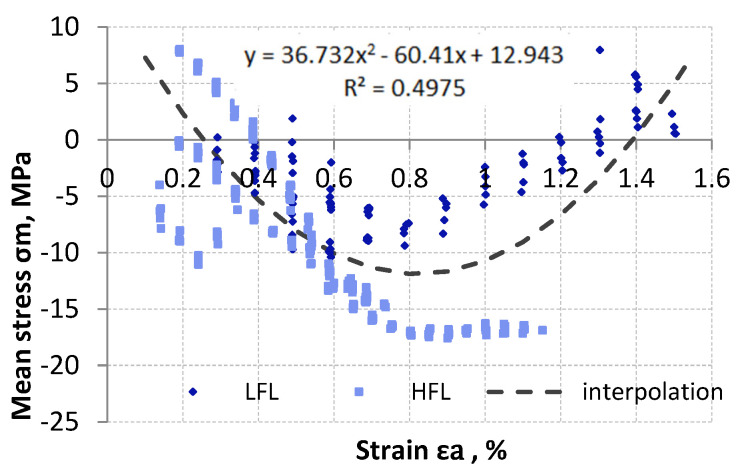
The curve of the mean stress relaxation as a function of the amplitude control strain.

However, when analyzing the mean stress as a function of strain amplitude, it can be observed that up to a certain strain value, the mean stress decreases with increasing strain. This situation is observed up to a strain value of about 0.7–0.9% for both tests performed (LFL and HFL). For the tested material subjected to variable loads, the specified strain may turn out to be a threshold value for which the relaxation direction reverses. The change in direction sense was particularly evident for tests with a large strain range, i.e., for LFL tests (Figure 6). Due to the similar nature of the behavior of the results of the mean stress value as a function of strain amplitude for both LFL and HFL, they were approximated by a single quadratic function equation (Figure 6).

### 3.3. Analysis of Hysteresis Loop Parameters

**Figure 7:**
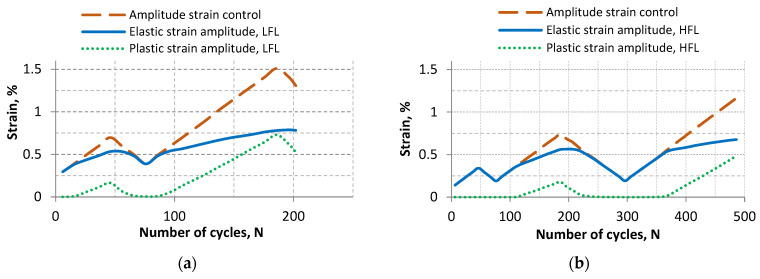
Summary of plastic and elastic strain as a function of the number of cycles: (**a**) LFL and (**b**) HFL.

Plastic and elastic strain curves determined from hysteresis loops as a function of increasing number of cycles were analyzed (Figure 7). A distinct plastic strain (a value of about 0.01%) appeared at a control strain amplitude of ε_a_ ≈ 0.39%. For LFL, the value of this plastic strain appeared at the first step of loading, while for HFL at the third step of loading, as it was only in the third step that the stress amplitude exceeded the yield stress value.

Once the stress amplitude corresponding to the yield stress (step II for LFL and step IV for HFL) is exceeded, distinct plastic strain (a value of about 0.01%) was recorded for a decreasing cycle only at a control variable of 0.45% for LFL and 0.49% for HFL. Subsequent increasing cycle resulted in the appearance of distinct plastic strain at an even higher control variable. Distinct plastic strain appeared at a control variable of 0.5% for LFL and 0.54% for HFL. The plastic strain mechanisms occurring during both increasing and decreasing cycles, once the value of the stress amplitude equivalent to the yield stress is exceeded, affect the cyclic hardening of this material.

**Figure 8:**
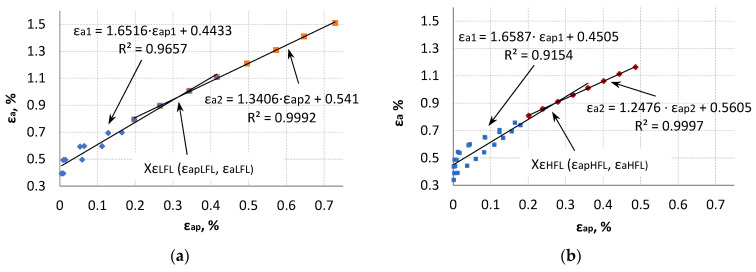
The control strain amplitude as a function of the plastic strain amplitude at LFL (**a**) and in HFL (**b**).

The graph showing the dependence of the control strain amplitude as a function of the plastic strain amplitude (Figure 8) is divided into two parts—the left and the right one. The left parts of Figure 8 are described by the straight line ε_a1_ = a·ε_apl_ + b, while the right parts of the graph are described by the straight line ε_a2_ = a·ε_ap2_ + b. It is observed that the slopes of the straight lines εa1 and ε_a2_ for LFL and HFL have similar values. The intersection of these straight lines is described by the point Xε (ε_ap_, ε_a_). The coordinates of these points are also similar and are (0.3135, 0.9612) and (0.2676, 0.8944) for LFL and HFL, respectively. Thus, taking into account the entire life of the tested items and looking at the formulas of the obtained straight lines, it can be concluded that, independent of the fatigue life result (which depends mainly on the value of the control amplitude), the selected value of the control variable will correspond to a specific plastic strain value comparable for the LFL and HFL tests.

The left side of the graphs corresponds to the results of tests with increasing/decreasing steps, while the right side corresponds mainly to the increasing step, which ended with the fracture of specimens. Experimental points to the left of point Xε and under the straight line ε_a1_ are mainly from the first loading steps, while those above straight line ε_a1_ are from subsequent loading steps. Subsequent loading cycles enforced by the control strain result in the fact that for the same values of the control variable ε_a_, the values of plastic strain ε_ap_ are smaller, which confirms the hardening of the material under cyclic loading.

### 3.4. Cyclic Modulus

**Figure 9:**
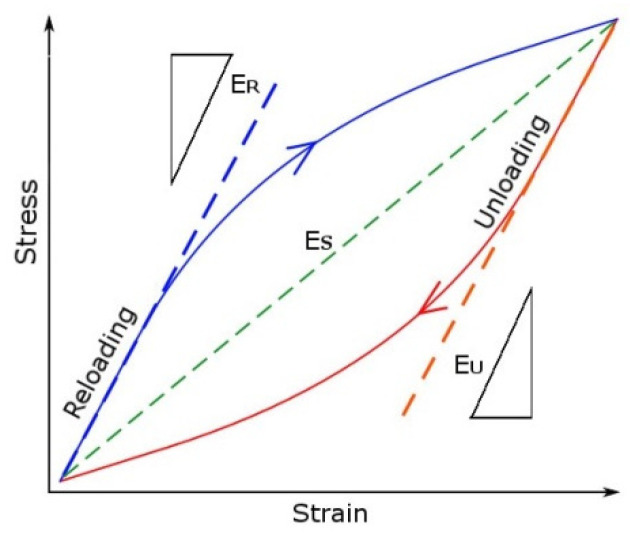
The diagram for determining cyclic moduli [22].

**Figure 10:**
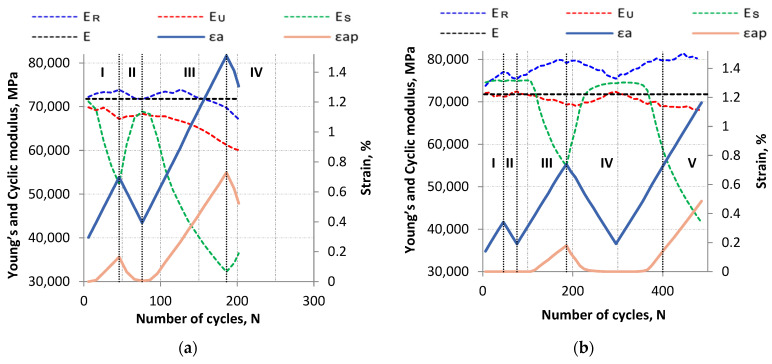
Summary of E, E_R_, E_U_, and E_S_ moduli values as a function of increasing number of cycles for (**a**) LFL and (**b**) HFL.

The cyclic modulus for tension loop branch (E_R_) and the compression loop branch (E_U_) was analyzed for the LFL and HFL tests. The cyclic module determined for the straight line connecting the edges of the hysteresis loop (E_S_) was also analyzed. The cyclic moduli were determined in accordance with the diagram shown in Figure 9. On the other hand, Figure 10 presents the summary of cyclic moduli as a function of the increasing number of cycles N. For comparison, Figure 10 also presents the Young’s modulus E as the mean value of the modulus obtained from the monotonic tensile test for the AA2519 alloy, derived from three publications [33,35,36].

In the first cycles of fatigue loading, especially when the yield stress point has not been exceeded, the cyclic modulus E_S_ should be characterized by a similar value to Young’s modulus E. Such a pattern is observed for the LFL test, while in the case of the HLF test, the cyclic modulus E_S_ is literally several percent higher than the modulus E. This difference, however, is at an acceptable level and may be related to the difference in material properties of the specimens as well as measurement errors.

The cyclic modulus E_S_ tends to decrease during a cyclically increasing value of the control strain. In case the control strain cyclically decreases, E_S_ modulus cyclically increases (Figure 10). Exceptions to this are life periods where the plastic strain value is close to (less than 0.01%) or equal to zero. In this situation, E_S_ modulus is relatively stable (at the beginning of fatigue life and the transition from step II to step III for LFL, and at step I, II, the beginning of step III, and the middle part of step IV for HFL). However, it should be noted that E_S_ value for LFL after increasing and decreasing cycles decreased from 71,188 MPa to 68,892 MPa, i.e., more than 3%, while for HFL it decreased from 75,120 MPa to 74,598 MPa, or less than 0.7%.

The nature of the change in the other cyclic moduli (E_R_ and E_U_) as a function of the number of cycles is similar for both LFL and HFL tests. In general, the E_R_ module increases when the control value increases cyclically, while it decreases when the control value decreases. For the E_U_ module, the situation is opposite. Such behavior of cyclic moduli, especially the E_R_ modulus, is observed until a certain period of fatigue life. In the case of the LFL test, this is about 126 cycles while for the HFL test it is about 446 cycles. The E_R_ modulus begins to decrease after this number of cycles. It is generally believed that the value of the classic Young’s modulus tends to decrease under the influence of cyclic loading. This is due to the fact that cyclic loading induces fatigue processes in the material, which in the initial steps lead, among other things, to microscale damage (cracks, internal structure changes) and thus to a decrease in the stiffness of the tested items. Such fatigue phenomena change the nature of the E_R_ modulus behavior causing a cyclic decrease in its value from a certain fatigue life value until complete failure of a specimen.

**Figure 11:**
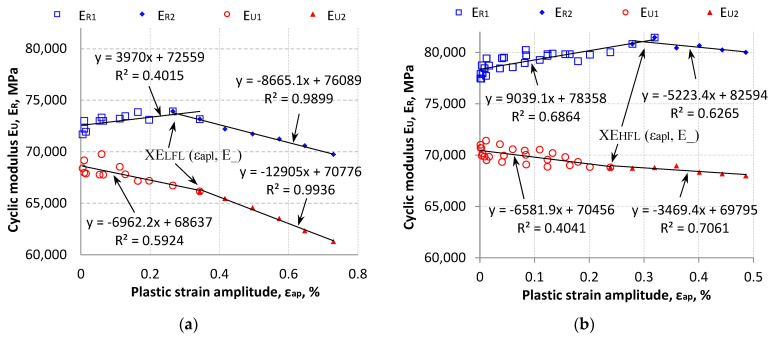
Summary of cyclic moduli E_R_ and E_U_ as a function of plastic strain amplitude ε_ap_: (**a**) for LFL and (**b**) for HFL.

The dependence of cyclic moduli (E_R_ and E_U_) as a function of plastic strain amplitude ε_ap_ was presented in the following analysis (Figure 11). The results of each cyclic modulus were described by two linear regressions, and the intersection of these regression lines was denoted by the XE_LFL/HFL_ (ε_ap__, E_R/U_) point. For the LFL test, the intersection points for E_R_ and E_U_ were XE_LFL___R_ (0.2794%, 73,668.1 MPa) and XE_LFL___U_ (0.3599%, 66,131.1 MPa), respectively. While for the HFL test, the intersection points for E_R_ and E_U_ were XE_HFL___R_ (0.2970%, 81,042.6 MPa) and XE_HFL___U_ (0.2124%, 69,058.2 MPa), respectively.

XE_LFL/HFL_ points marked in Figure 11 are indicative of the change in material properties under cyclic loading. To the left of points XE_, the values of the cyclic moduli E_R1_ for the conducted tests increase with increasing plastic strain ε_ap_. Then, after passing the characteristic XE_ points, the values of cyclic moduli E_R2_ decrease with the increase in plastic strain ε_ap_, while cyclic moduli E_U_ (both to the left and to the right of the XE_ points) tend to decrease throughout the entire tested period.

In order to use the cyclic modulus in the following part of the paper to analytically describe cyclic material properties, the mean values of cyclic moduli were calculated. The mean value was calculated for the values of cyclic moduli to the left of the XE_LFL_ and XE_HFL_ points. It was noted that these values resulted in the best fit of the analytical model to the obtained study. Mean values were determined for the results of moduli E_R1_ (by determining E_R1m_) and E_U1_ (by determining E_U1m_) separately for the LFL and HFL test. Then the mean cyclic modulus E_m__ = (E_R1m_ + E_U1m_)/2 was determined. The average values of E_m_LFL_ for LFL and E_m_HFL_ for HFL were 70,478 MPa and 74,298 MPa, respectively. These values are presented in Table 2.

### 3.5. Cyclic Yield Strength

**Figure 12:**
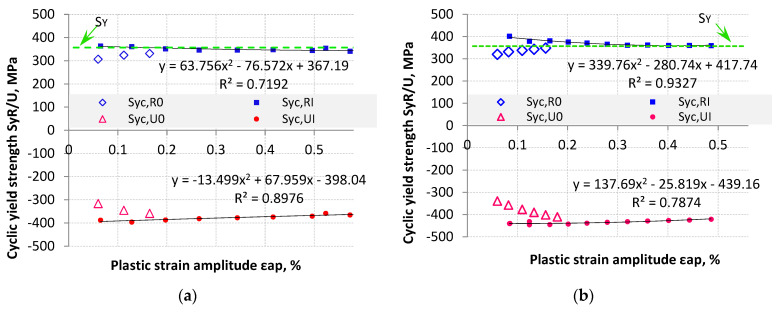
Summary of S_YC_ (S_YC,R_ and S_YC,U_) as a function of plastic strain amplitude ε_ap_: (**a**) for LFL and (**b**) for HFL.

The cyclic yield strength (S_YC_) was calculated for the increasing (S_YC,R_) branch loop and decreasing (S_YC,U_) branch loop. The S_YC_ was calculated for the offset value of 0.2%. The S_YC_ was calculated for hysteresis loops with plastic strain amplitude equal to and greater than 30% of the offset value (ε_ap_ ≥ 0.06%). The values of S_YC,R_ and S_YC,U_ for LFL and HFL, as a function of plastic strain amplitude ε_ap_, are shown in Figure 12a for the LFL test and Figure 12b for the HFL test.

The results of S_YC_ from the first loading blocks were marked with the subscript “0” (S_YC,R0_ and S_YC,U0_), while those from blocks where the S_YC_ value stabilized with “I” (S_YC,RI_ and S_YC,UI_). Lower absolute S_YC_ values were recorded for the first loading blocks as compared to the later loading blocks. This proves that for this type of material S_YC_ limit tends to harden cyclically. The hardening reached almost 30% over the lowest cyclic yield strength. S_YC_ results from the first loading blocks were discarded in the statistical analysis. The results from subsequent loading blocks S_YC,RI_ and S_YC,UI_ were described by a quadratic polynomial regression function, which yielded a coefficient of determination (R^2^) of 0.71−0.93.

The stabilized absolute S_YC_ value for both the LFL test (Figure 12a) and HFL test (Figure 12b) results decreased with increasing plastic strain amplitude ε_ap_. The differences in the determined S_YC_ value as a function of plastic strain amplitude were not significant and were at most about 10%. The absolute mean values of the stabilized S_YC_ were S_YC,R_ = 322 MPa and S_YC,U_ = 344 MPa for the LFL test, and S_YC,R_ = 373 MPa and S_YC,U_ = 434 MPa for the HFL test. The mean value of cyclic tensile yield strength (S_YC,R_) was lower than the mean value of cyclic compressive yield strength.

The mean value of cyclic tensile yield strength (S_YC,R_) was also determined for all previously analyzed points for both the LFL test and HFL test. This value was 354 MPa. It should be noted that this value is comparable to the yield strength calculated for the monotonic tensile test S_Y_ = 353 MPa. The S_Y_ is also plotted in Figure 12 to illustrate the results.

### 3.6. Fatigue Fracture Surface

**Figure 13:**
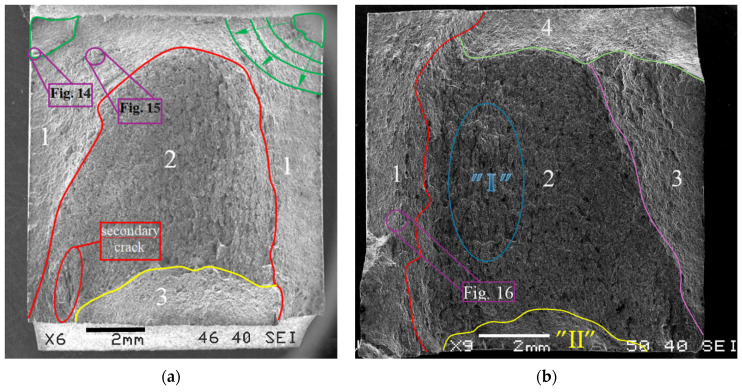
Marked fracture zones for test specimens from (**a**) LFL and (**b**) HFL tests.

A scanning electron microscope (SEM) was used to take photographs of specimen fractures, taking a series of photos at various magnifications. Several characteristic fracture zones were identified in the photos taken. Three zones were identified for the LFL test fracture (Figure 13a) and four zones were identified for the HFL test fracture (Figure 13b). It should be noted that the number of distinct zones depends on the image filtering method (SEM, SEI, REF).

**Figure 14:**
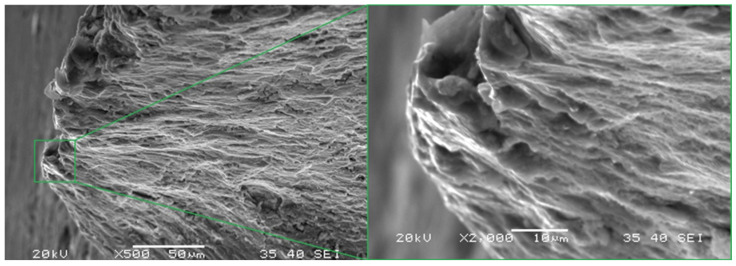
Marked part of the LFL test specimen in which the crack focus was found.

**Figure 15:**
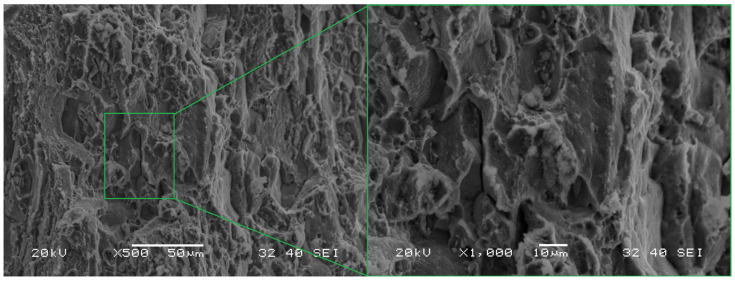
Marked part of the LFL test specimen with a visible secondary crack.

Two main fracture foci were identified for the LFL test fracture (marked green in Figure 13a). A detailed analysis of one of them made it possible to identify the crack initiation point, which formed the river marks structure during propagation, as shown in Figure 14. This fracture is topographically complex. One of the most characteristic features of the fracture is a large secondary crack, a detail of which is shown in Figure 15.

No distinct fracture foci are visible in the HFL test fracture (Figure 13b). The cracking process most likely began at the edge, which in the photograph shown is the lower edge of the specimen (marked “II”). The relatively complex nature of the crack is observed in the area “I” (forming part of zone 2), which is the result of intercrystalline cracks propagating in multiple directions of which the main direction was from the lower to the upper edge of the specimen propagating in the direction of the load. As a result, the direction of the main crack was neither parallel nor perpendicular to the plate rolling direction. The characteristic jagged structure is the result of exactly this propagation.

**Figure 16:**
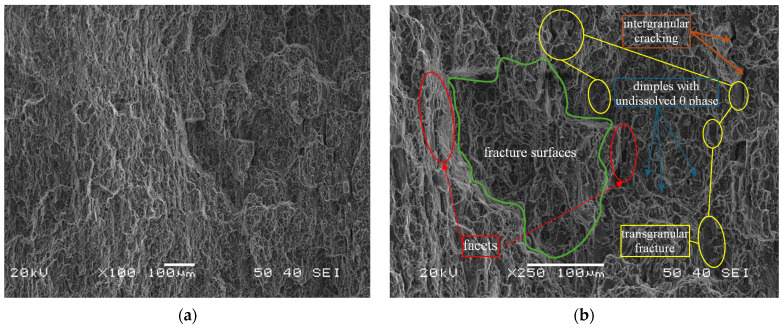
A characteristic part of the specimen fracture from the HFL test (**a**) and a description of this part at a higher magnification (**b**).

**Figure 17:**
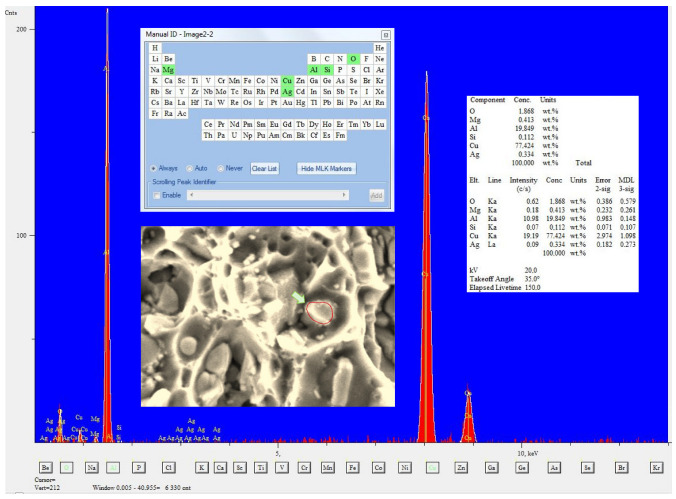
Chemical composition measured at the bottom of the dimple transverse to the spherical undissolved theta phase particle with marked area where the chemical composition analysis was performed.

The fracture of the selected fragment of the HFL test specimen (Figure 16) is of transcrystalline plastic character with sparse intercrystalline cracks present. A feature common to the analyzed fracture in this area is the occurrence of characteristic faults penetrating deep into the material. They most likely occur between individual layers of the rolled plate. Facets are visible on the walls of these cracks, and (at slightly higher magnifications) voids and micro-cracks are discernible. Particles of undissolved θ-phase are visible at the bottom of the dimples, as evidenced by the result of the chemical composition analysis of the fragment containing the said particles, the spectrum of which is shown in Figure 17. Some of them have kept their original spherical shape, while some have cracked (Figure 17).

### 3.7. Cyclic Stress–Strain Curve (CSS)

**Figure 18:**
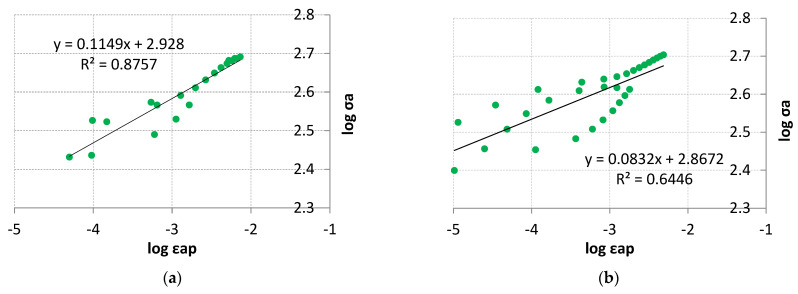
Stress amplitude logarithms versus plastic strain amplitude logarithms for (**a**) LFL and (**b**) HFL.

As demonstrated in the above analysis, the tested material has cyclically unstable material properties. At lower levels of the control variable, periods of cyclic material weakening prevail, while at higher levels of the control variable, periods of cyclic hardening predominate. Due to this, describing the material properties through conventional analytical equations such as the Ramberg–Osgood model using n’ and K’ [9,10] becomes significantly more difficult or impossible. Material data developed in this way in order to describe material properties may lead to differences between actual material properties. Figure 18 shows an attempt to describe experimental data from the LFL test (Figure 18a) and the HFL test (Figure 18b) with a straight line as a function of logσ_a_ of the variable logε_ap_.

The description of the experimental results with a linear regression function (Figure 18) results in a relatively high coefficient of linear determination R^2^ = 0.8757 especially when it comes to the linear correlation of the LFL test results. A much worse coefficient of linear determination, R^2^ = 0.6446, was obtained for the linear correlation of the HFL test results. The n′ and K′ coefficients were determined for the LFL and HFL test results, which are summarized in Table 2.

An attempt was also made to describe the test results using two linear correlations. The value of ε_apX_ calculated from the mean value derived from the intersection points of Xε_LFL/HFL_, XE_LFL/HFL___R_, and XE_LFL/HFL___U_ was used as a criterion for dividing the experimental test results. These values amounted to ε_apXLFL_ = 0.2382% for the LFL test and ε_apXHFL_ = 0.1943% for the HFL test, respectively.

**Figure 19:**
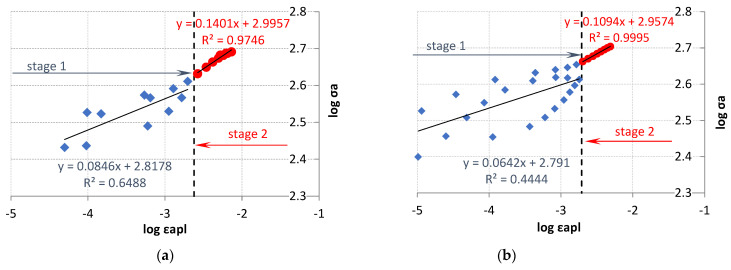
Stress amplitude logarithms versus plastic strain amplitude logarithms for (**a**) LFL and (**b**) HFL after splitting the test results into two parts.

For the criterion adopted in such a way, the experimental results were divided into two parts: stage 1 and stage 2 (Figure 19). A very high coefficient of determination was obtained for stage 2. Such a high coefficient of determination was not obtained for the results in the first part of the chart (stage 1). This is due to the fact that the test results in the first part of the chart (stage I) come from both increasing and decreasing steps, which, with cyclically unstable materials, affects their scattering. The coefficients n_1_′, n_2_′, K_1_′, and K_2_′ were determined for the stage 1 and stage 2 test results. The values of these factors are summarized in Table 2.

**Figure 20:**
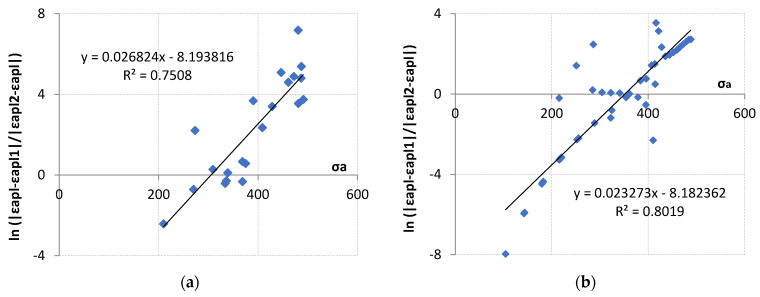
Graphical interpretation of the determination of the material parameters A and B for (**a**) LFL test and (**b**) HFL test.

A formula shown in [32], used to describe the tensile curve, was also proposed for the analytical description of the presented test results. The present formula 1 is a slight modification of that originally proposed in [31]. This formula takes into account A and B constants, described by Equation (1). The coefficients n_1_′ and K_1_′ were used to determine plastic strain amplitude ε_ap1_, while the coefficients n_2_′ and K_2_′, listed in Table 2, were used to determine ε_ap2_. A graphical interpretation of the results obtained from Equation (1) is shown in Figure 20. Material parameters A and B are summarized in Table 2. A high coefficient of determination was obtained for the analyzed results, the value of which exceeded 0.75 in both cases.

(1) $$ln\frac{\left|\varepsilon apl-\varepsilon a{pl}_{1}\right|}{\left|\varepsilon a{pl}_{2}-\varepsilon apl\right|}=A\cdot \sigma a+B$$

**Figure 21:**
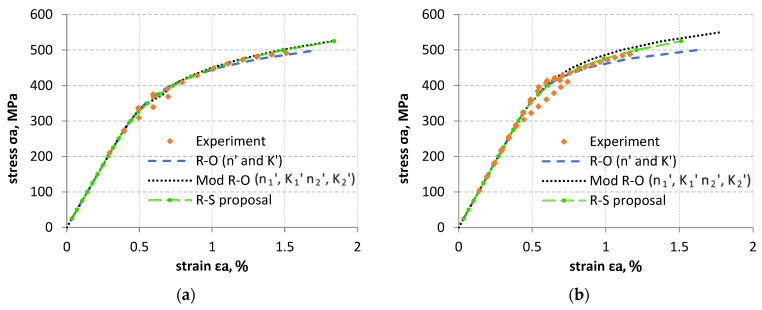
Graphical interpretation of the mathematical description of the LFL (**a**) and HFL (**b**) test results using the Ramberg–Osgood (R–O) model, the R–O modification (Mod R–O) and Soltysiak’s proposal (R–S proposal).

The parameters summarized in Table 2 allowed to describe the obtained test results using three analytical models. The first description was based on the Ramberg–Osgood Equation (2), where the cyclic mean modulus E_m_ was used instead of the standard Young’s modulus. On the other hand, the second description was based on division of the Ramberg–Osgood curve into two parts (Equation (3)) according to the idea of determining K_1_′, K_2_′, n_1_′, and n_2_′ coefficients. For the last description, Soltysiak’s proposal 4 [32], based on a slight modification of the formula originally introduced by Li [31], was applied. This description was previously used to describe monotonic properties. Graphical interpretation of these descriptions is shown in Figure 21.

(2) $$\begin{array}{cc} \mathrm{For the whole range:} & \varepsilon_{a}=\varepsilon_{ae}+\varepsilon_{apI}=\frac{\sigma }{E_{m}}+{\left(\frac{\sigma }{K^{\prime }}\right)}^{\frac{1}{n^{\prime }}} \end{array}$$

(3) $$\begin{array}{cc} \mathrm{For stage 1:} & \varepsilon_{a1}=\varepsilon_{ae}+\varepsilon_{ap1}=\frac{\sigma }{E_{m}}+{\left(\frac{\sigma }{K_{1}^{\prime }}\right)}^{\frac{1}{n_{1}^{\prime }}}\;\;\;\;\;\;\;\;\;\;\;\;\;\mathrm{for}\;\varepsilon_{ap}\leq \varepsilon_{apXLFL}\;or\;\varepsilon_{apXHFL}\\ \mathrm{For stage 2:} & \varepsilon_{a2}=\varepsilon_{ae}+\varepsilon_{ap2}=\frac{\sigma }{E_{m}}+{\left(\frac{\sigma }{K_{2}^{\prime }}\right)}^{\frac{1}{n_{2}^{\prime }}}\;\;\;\;\;\;\;\;\;\;\;\;\;\mathrm{for}\;\varepsilon_{ap}>\varepsilon_{apXLFL}\;or\;\varepsilon_{apXHFL} \end{array}$$

(4) $$\begin{array}{cc} \mathrm{For the whole range:} & {\varepsilon_{a}=\varepsilon_{ae}+\varepsilon }_{ap}=\frac{\sigma }{E_{m}}+\frac{\varepsilon_{ap2}\cdot \exp\left(A\sigma +B\right)+\varepsilon_{ap1}}{1+exp\left(A\sigma +B\right)},\;\varepsilon_{ap1}{=\left(\frac{\sigma }{K_{1}^{\prime }}\right)}^{\frac{1}{n_{1}^{\prime }}},\;\varepsilon_{ap2}{=\left(\frac{\sigma }{K_{2}^{\prime }}\right)}^{\frac{1}{n_{2}^{\prime }}} \end{array}$$

**Figure 22:**
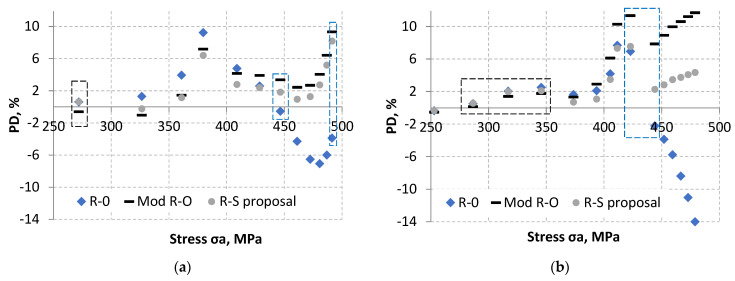
Graphical interpretation of the percentage strain difference determined from analytical models in relation to experimental strain results for the LFL test (**a**) and HFL test (**b**).

When analyzing Figure 21, it is difficult to identify the model that best describes the experimental tests. In order to compare the models presented, the formula (5) was used to determine the percentage difference from the experimental results (PD, %). For several experimental points for a given value of experimental strain, the value of stress σ_a_ and strain ε_a_ were determined as the mean value. A perfect fit of the model to the experimental results means PD = 0%. Its graphical interpretation is shown in Figure 22.

(5) $$PD=\frac{\varepsilon_{a}-\varepsilon_{ai}}{\varepsilon_{a}}\cdot 100\%$$

where ε_ai_—total strain amplitude determined from individual R–O, Mod R–O, R–S proposal models, respectively.

The best model fit to experimental results for both the LFL and HFL test was obtained for the R–S proposal model. However, not all experimental points were better fitted using the R–S model. These points are marked in Figure 22 with black and blue rectangles. The black rectangles indicate data for which the experimental points were slightly better described by the R–O modification. The blue rectangles indicate data for which the experimental points were slightly better described by the R–O model.

## 4. Conclusions

The article presents an analysis of the outcomes for AA2519 aluminum alloy exposed to variable loads. The variable loads were implemented with a strain control program. One test was carried out with higher strain control resulting in lower fatigue life (LFL test), while the other involved testing at lower strain values, resulting in higher fatigue life (HFL test). The test results obtained were described by several analytical models. As a result of the analysis, the following conclusions have been reached.

The tested material is characterized by cyclic hardening, which was evident at individual levels of the control variable ε_a_, especially in the strain range of 0.5–1.3%, where a cyclic increase in the stress amplitude predominated. This is also evidenced by the maximum values of stress amplitude recorded for LFL and HFL tests, which are 3.4% and 2.8% higher than the ultimate strength of the tested material, respectively.

A change in the direction of mean stress relaxation as a function of the control strain was observed. When the control strain amplitude increased to approximately 0.8%, the mean stress value decreases, but above this value, it begins to increase again. The relaxation of the mean value towards a positive value at such high strain values may be caused by the appearance of cracks visible in certain areas of fractures, which are large stress concentrators and are accompanied by large strain necessary to achieve forced control strain. These processes can decrease the compression strength of the material and increase its tension strength (harden it).

The intersection points of linear correlations describing cyclic moduli as a function of the plastic strain amplitude and linear correlations of the control strain amplitude as a function of the plastic strain amplitude can become a criterion describing the change in material properties for selected cyclically unstable materials and such materials that are not loaded using multiple steps with increasing/decreasing strain.

Analysis of the fractures of the LFL and HFL test specimens showed that they were similar, although differences were also found. The similarities include the complex topographic structure of the fracture. In both cases, the fractures included a zone of the crack at the ultimate tensile strength. The differences, on the other hand, included primary cracks and one visible secondary crack within the entire volume of the material, found in the fracture of the LFL test specimen. It should also be stressed that when analyzing the fracture of the LFL test specimen, the dimples were more difficult to see than in the HFL test specimen. The set of fracture features indicates that the LFL test specimen was subjected to higher plastic load virtually until the end of the fatigue life and may have temporarily weakened during the experiment due to a reduction in cohesion forces within the material resulting from secondary cracks. The HFL test specimen, on the other hand, did not show this feature, which indicates that in the final phase of the experiment the material reached cyclic equilibrium.

The mean value of cyclic yield strength for tensile cycles is comparable to that of tensile yield strength. Therefore, it can be assumed that the yield strength value derived from the monotonic tensile test does not introduce significant error into the characterization of cyclic properties. However, it should be noted that the mean value of cyclic tensile yield strength is lower than the mean value of cyclic compressive yield strength, which may be related to mean stress relaxation.

The proposed material model, which is based on material constants A and B to describe cyclic properties of the material and the cyclic modulus E_m_ calculated from the first stage demonstrates good compliance with experimental results. Moreover, the proposed model enables the description of material properties using a single equation which facilitates its application in engineering analyses.

## Figures and Tables

**Table 1:** Chemical composition and mechanical properties of AA2519 aluminum alloy [33,34].

Chemical Composition (%)									
Si	Fe	Cu	Mg	Zn	Ti	Sc	Zr	V	Al
0.06	0.08	5.77	0.18	0.01	0.04	0.36	0.12	0.12	balance
**Tensile properties**									
Sy (Rp_0.2_)		Su (Rm)		E				A_5_	
MPa		MPa		GPa				%	
353		475		67.5 [33], 78.0 [35], 69.9 [36]				16.3	

**Table 2:** Cyclic stress–strain curve parameters.

Test	Em, MPa	n′	K′	n_1_′	K_1_′	n_2_′	K_2_′	A	B
**LFL**	70,478	0.1149	847.14	0.0846	657.39	0.1401	990.21	0.026824	−8.193816
**HFL**	74,298	0.0832	736.52	0.0642	618.02	0.1094	906.47	0.023273	−8.182362

## Data Availability

The data supporting the findings of this study are contained within the article. Further inquiries can be directed to the corresponding author.
